# Sensitivity of the stripe-faced dunnart, *Sminthopsis macroura* (Gould 1845), to the insecticide, fipronil; implications for pesticide risk assessments in Australia

**DOI:** 10.1007/s10646-022-02549-z

**Published:** 2022-05-05

**Authors:** Paul G. Story, Lyn A. Hinds, Steve Henry, Andrew C. Warden, Greg Dojchinov

**Affiliations:** 1 Australian Plague Locust Commission, Department of Agriculture, Water and Environment, Canberra, ACT 2601 Australia; 2 Commonwealth Scientific and Industrial Research Organisation, Health and Biosecurity, Black Mountain Laboratories, Acton, ACT 2600 Australia; 3 Commonwealth Scientific and Industrial Research Organisation, Land and Water, Black Mountain Laboratories, Acton, ACT 2600 Australia

**Keywords:** Dunnart, Acute oral toxicity, Fipronil, Median lethal dose, *Sminthopsis macroura*, Pesticide risk assessment

## Abstract

A lack of toxicity data quantifying responses of Australian native mammals to agricultural pesticides prompted an investigation into the sensitivity of the stripe-faced dunnart, *Sminthopsis macroura* (Gould 1845) to the insecticide, fipronil (5-amino-3-cyano-1-(2,6-dichloro-4-trifluoromethylphenyl)-4-trifluoromethylsulfinyl pyrazole, CAS No. 120068-37-3). Using the Up-And-Down method for determining acute oral toxicity in mammals (OECD) median lethal dose estimates of 990 mg kg^−1^ (95% confidence interval (CI) = 580.7–4770.0 mg kg^−1^) and 270.4 mg kg^−1^ (95% CI = 0.0–>20,000.0 mg kg^−1^) were resolved for male and female *S. macroura*, respectively. The difference between median lethal dose estimates for males and females may have been influenced by the older ages of two female dunnarts. Consequently, further modelling of female responses to fipronil doses used the following assumptions: (a) death at 2000 mg kg^−1^, (b) survival at 500 mg kg^−1^ and (c) a differential response (both survival and death) at 990 mg kg^−1^. This modelling revealed median lethal dose estimates for female *S. macroura* of 669.1 mg kg^−1^ (95% CI = 550–990 mg kg^−1^; assuming death at 990 mg kg^−1^) and 990 mg kg^−1^ (95% CI = 544.7–1470 mg kg^−1^; assuming survival at 990 mg kg^−1^). These median lethal dose estimates are 3–10-fold higher than available LD_50_ values of 94 mg kg^−1^ for a similarly sized eutherian mammal, *Mus musculus* (L. 1758) and 97 mg kg^−1^ for *Rattus norvegicus* (Birkenhout 1769). Implications for pesticide risk assessments in Australia are discussed.

## Introduction

Fipronil (5-amino-3-cyano-1-(2,6-dichloro-4-trifluoromethylphenyl)-4-trifluoromethylsulfinyl pyrazole, CAS No. 120068-37-3), a phenyl-pyrazole compound, is a broad spectrum, low dose chemical registered for use in many countries including Russia, South Africa and Australia (Balanca and de Visscher 1997; Bobe et al. 1998) and in 2013 was banned from use on corn and sunflower crops by the European Union based on its role in ‘colony collapse’ in bee populations in France (Bijleveld van Lexmond et al. 2014; Chagnon et al. 2014; Holder et al. 2018). This pesticide is an active neurotoxicant and is a potent disrupter of insect central nervous systems where it works by interfering with the passage of chloride ions through the chloride-gated channel regulated by gamma-aminobutyric acid (GABA) receptors (Hainzl et al. 1998) and acts via both direct contact and via-stomach action (Story et al. 2005).

Although fipronil is used throughout the world as a crop protection agent, little information exists concerning either its toxicological impacts on vertebrates, or what the ecological and population-level consequences of exposure might be (Smith et al. 2010). This data gap is problematic for the assessment of environmental risk associated with the use of fipronil for locust control where low-volume, oil-based insecticide formulations are used over arid and semi-arid native grasslands to control acridid (grasshopper and locust) populations in several countries including Australia (Story et al. 2005; Walker et al. 2016). The use of fipronil for acridid control in Africa has been discontinued largely due to its environmental impacts (Peveling et al. 1999, 2003; Steinbauer and Peveling 2011).

Mammalian risk assessments undertaken in Australia for fipronil cite only two LD_50_ values, making the development of species sensitivity distributions for a complete probabilistic risk assessment impossible (Posthuma et al. 2002). Furthermore, both estimates of acute oral toxicity are contained within industry reports listed as ‘Commercial in Confidence’ and so the complete details of the study parameters and results are not available through the established scientific literature. Rather, summaries by the United States Environmental Protection Agency (USEPA) need to be relied upon to gauge the potential mammalian responses to fipronil exposure (Food and Agriculture Organisation of the United Nations 1997). In one study, an LD_50_ estimate of 97 mg kg^−1^ has been reported for an unspecified rat species with abnormal gait and posture, piloerection, lethargy, tremors and convulsions all reported as signs of intoxication (Environment Australia 1998). To date, there are only two LD_50_ estimates for *Mus musculus* (L. 1758), a similar-sized eutherian mammal to dunnarts, namely 95 mg kg^−1^ (Tomlin 2006) and 94 mg kg^−1^ (Environment Australia 1998) and there are no studies quantifying the acute oral toxicity of fipronil for Australian endemic mammalian fauna.

Recent research has shown that two dunnart species (the fat-tailed dunnart, *Sminthopsis crassicaudata* (Gould 1844); and stripe-faced dunnart, *S. macroura)*, were 10–14 times more sensitive to the organophosphorus insecticide, fenitrothion (O,O-dimethyl-O-(3-methyl-4-nitrophenyl)-phosphorothioate), another locusticide (Story et al. 2011), compared to eutherian mammals. The inclusion of these median lethal dose estimates into species sensitivity distributions (SSD) during the development of an environmental risk assessment resulted in allowable residue values derived at the 5% protection threshold (HD_05_) being almost halved from 177 to 93.5 mg kg^−1^ (Story et al. 2011). While there is often a need to extrapolate from a narrow range of organisms tested under standard laboratory conditions to free-living populations or ecosystems during risk assessments (Barnthouse et al. 2008; van Straalen 2002), differences in the abovementioned hazard thresholds derived by including acute oral toxicity values for dunnarts (Story et al. 2011), highlights the need to evaluate effects of pesticides on non-target species, particularly when these species are phylogenetically distinct from those originating in North America or the European Union (Story et al. 2011).

**Fig. 1:**
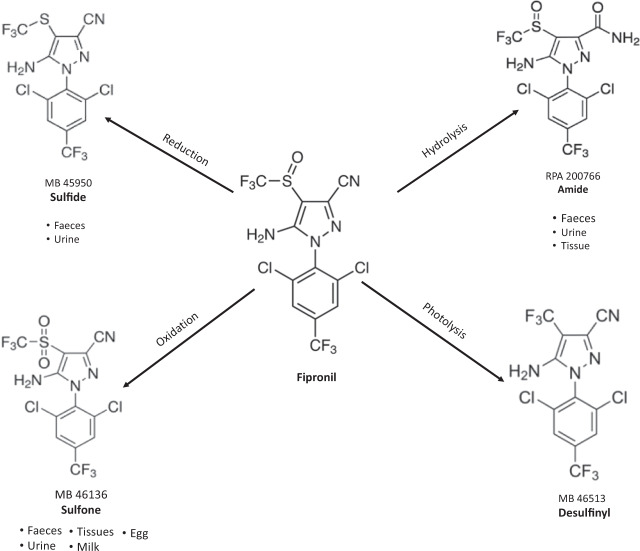
Metabolism of fipronil in vertebrates with the addition of the photolysis degradation pathway (adapted from Tingle et al. (2000) and Kitulagodage (2011))

Research into the impacts of fipronil exposure on avian species has shed light on the importance of toxicological testing on a broader range of species than those currently presented in pesticide registration evaluations (Smith et al. 2010). Previously, acute fipronil toxicity was only considered of concern in the Galliformes (Tingle et al. 2000). However, in more recent research, fipronil’s avian acute oral toxicity has been shown to group by avian order when additional species are tested (Kitulagodage 2011). Moreover, it has been shown that (a) pesticide adjuvants add synergistically to the overall toxicity of formulations (Kitulagodage et al. 2008), (b) the metabolic fate of fipronil closely resembles that of organochlorine insecticides (OC) (Kitulagodage et al. 2011b) and (c) that both fipronil and its oxidative metabolite fipronil-sulfone (Fig. 1, hereafter referred to as fip-sulfone), can be maternally transferred resulting in developmental abnormalities in hatchlings (Kitulagodage et al. 2011a).

In the current study the Up-And-Down protocol (UDP), devised by the Organisation for Economic Cooperation and Development (OECD) was used to quantify the median lethal dose of fipronil to *S. macroura*. This technique is a useful alternative to conventional LD_50_ testing (Bruce 1985) and has been used in previous studies to quantify the acute oral toxicity of another locusticide, fenitrothion, to the marsupials, *S. crassicaudata* and *S. macroura* (Story et al. 2011). Although enabling median lethal dose estimates to be quantified for a given species, the UDP technique has been shown to be comparable to conventional toxicity testing methodologies while requiring far fewer animals to resolve an estimate (Lipnick et al. 1995), thereby allowing a comparison of pesticide sensitivity of Australian marsupial fauna derived here with non-native eutherian mammals tested elsewhere (Story et al. 2011).

The combination of a significant overlap in habitat preferences between the Australian plague locust (*Chortoicetes terminifera* Walker 1870) and *S. macroura*, small body mass (some as low as 7 g (van Dyck and Strahan 2008)), high metabolic requirements, their primarily insectivorous diet and the ability to gorge feed on pesticide-exposed locusts, make these species particularly vulnerable to the effects of chemically-based locust control (Story 2015). Consequently, members of the Dasyuridae are the taxa most likely, of the numerous mammal species in Australia, to be affected by pesticide exposure resulting from locust spray operations (Story et al. 2016).

This study quantifies the acute oral toxicity of fipronil to the endemic metatherian Australian mammal, *S. macroura* and compares the values obtained with the very limited data available for mammals more broadly. Pesticide residue levels of the parent compound, fipronil and its metabolites in plasma, brain, liver, kidney and caudal and subcutaneous adipose tissues were also quantified from dunnart tissue to serve as a pilot investigation for a subsequent study into the comparative metabolic fate of fipronil in two similar-sized but systematically divergent species, *M. musculus* (eutherian) and *S. macroura* (metatherian). Implications for pesticide risk assessments in Australia are discussed.

## Materials and methods

### Animal housing

Dunnarts used in the trial were sourced from a breeding colony kept at Commonwealth Scientific and Industrial Research Organisation (CSIRO) Black Mountain Laboratories (Acton, Australian Capital Territory, Australia) made up of either field-collected animals (*n* = 9) or first-generation descendants of those individuals (*n* = 9). All dunnarts were sexually mature at the time of the experiment and were maintained in individual cages on a day: night cycle that reflected ambient Canberra conditions during May–August 2016 and kept at a constant temperature of 23 °C. Dunnarts were fed low-fat minced beef, supplemented with calcium carbonate (25 g kg^−1^) and 0.015% potassium iodide solution (43 mL per 12 kg lean beef mince) as used by previous authors to maintain *S. macroura* colonies (Selwood and Cui 2006). Water was available ad libitum. Dunnarts were fasted for 24 h before the administration of fipronil doses and then observed using video recording for 48 h after pesticide exposure and then daily without video recording for the following 12 days. Food was returned to the dunnarts’ cages 24 h after dosing.

### Determination of acute oral toxicity

In total, 18 dunnarts (7 males and 11 females) were used to determine the acute oral toxicity of fipronil with doses administered according to the UDP dosing schedule. Each animal was weighed immediately prior to dosing and doses were made up using reference grade fipronil (ChemService Inc. West Chester, PA, USA; CAS number: 120068-37-3, Lot number: 3719000), dissolved in 20 μL of acetone made up to 0.2 mL using canola oil. Each dose was given oesophageally using a 23-gauge gavage needle attached to a 1 mL syringe.

We followed OECD Guideline 425 (Organisation for Economic Cooperation and Development 2001) to estimate the acute oral toxicity value, in this case a median lethal dose along with its corresponding confidence interval for each gender. We used the Main Test of this guideline, with an alpha value (*α*) of 0.25 and a starting dose of 175 mg kg^−1^. The UDP protocol stipulates that where no estimate of the substance’s lethality is available, dosing should be initiated at 175 mg kg^−1^. In most cases, this dose is sublethal and therefore serves to reduce the level of pain and suffering experienced by animals used in the experiment.

The UDP dosing protocol consists of a single-ordered dose progression in which animals are dosed individually and then observed for a minimum of 48 h before a subsequent dose is administered to another animal. If a dunnart survived the dose given to it within this short-term interval, the next animal received a higher dose, but if an animal succumbed to dosing within this time period, the dose progression proceeded with a lower dose (see Tables 1 and 2) as prescribed in the Acute Oral Toxicity (AOT) software programme (Organisation for Economic Cooperation and Development 2001) used for the analysis of dosing data. The long-term fate of dunnarts, defined here as the fate of animals at 14 days post exposure surviving a given dose of fipronil, was also recorded. Dosing continued until one of the three standard stopping criteria was met:

- three consecutive animals survived at the upper bound of dosing,
- five reversals occurred in any six consecutive animals tested (when a reversal is created by a pair of responses in a situation in which a nonresponse is observed at a particular dose and a response is observed at the next dose tested, or vice versa),
- or at least four animals have followed the first reversal and the specific likelihood ratios exceed the critical value as determined by the AOT software.

**Table 1:** Dose progression for Up-And-Down protocol given with short-term (48 h) and long-term (14 days) fates of individual male *Sminthopsis macroura* dosed orally with fipronil and time to death for those dunnarts encountering a lethal dose

Test animal	Estimated age at time of dosing (months)	Dose (mg kg^−1^)	Short-term fate (48 h)	Long-term fate (14 days)	Time to death (hh:mm)
014	30	175	O^a^	O	
015	30	310	O	O	
023	30	550	O	O	
026	30	990	X^b^	X	00:23
033	18	550	O	O	
036	18	990	O	O	
038	18	2000	X	X	12:39

^a^O = Survival at the given dose

^b^X = Death at the given dose

**Table 2:** Actual and modelled^a^ dose progressions for Up-And-Down protocol given with short-term (48 h) and long-term (14 days) fates of individual female *Sminthopsis macroura* dosed orally with fipronil

Test animal	Estimated age at time of dosing (months)	Dose (mg kg^−1^)	Actual dose progression^b^			Modelled dose progression, excluding animals 016 and 001, with *HH* surviving dose of 990 mg kg^−1c^		Modelled dose progression, excluding animals 016 and 001, with *HH* dying at a dose of 990 mg kg^–1d^	
			Short-term fate (48 h)	Long-term fate (14 days)	Time to death (hh:mm)	Short-term fate (48 h)	Long-term fate (14 days)	Short-term fate (48 h)	Long-term fate (14 days)
016	>36	175	X	X	37:00				
001	>30	99	X	X	23:16				
032	18	55	O	O		O	O	O	O
031	18	99	O	O		O	O	O	O
035	18	175	O	O		O	O	O	O
040	18	310	O	O		O	O	O	O
045	18	550	O	O		O	O	O	O
046	18	990	O	X	105:37	O	X	O	X
051	18	2000	O	X	64:24	O	X	O	X
054	18	2000	X	X	01:35	X	X	X	X
060	8	990	X	X	39:45	X	X	X	X
*GG*^d^		550				O	O	O	O
*HH*		990				O	O	X	X

^a^Modelled dose progressions assume survival at 550 mg kg^−1^, death at 2000 mg kg^−1^ and develop two scenarios (see ^c^ and ^d^ above) reflecting the differential response of dunnarts at a dose of 990 mg kg^−1^

^b^X = Death, O = Survival, at the given dose

^c^Modelled dose progression, excluding the first two female dunnarts used and including additional hypothetical animals (*GG* and *HH*) with *HH* surviving the final dose of 990 mg kg^−1^

^d^Modelled UDP dose progression, excluding the first two female dunnarts used and including additional hypothetical animals (*GG* and *HH*) with *HH* succumbing to the final dose of 990 mg kg^−1^

After the stopping criteria had been reached, an estimate of the LD_50_ value (calculated as the median lethal dose using maximum likelihood statistics) and the associated confidence limits were calculated using the AOT software Statistical Program version 1.0 (Organisation for Economic Cooperation and Development 2001).

The body mass of each dunnart was measured approximately 30 min before pesticide exposure and then at daily intervals, up to 14 days thereafter for those dunnarts not incurring a lethal dose. Body mass data were analysed using *t*-tests on data pooled by dose for males and females (IBM SPSS 2017). Animals which became moribund were euthanased using isoflurane under oxygen and tissue samples collected and stored at −80 °C until subsequent analysis (see below).

### Quantification of tissue residue levels and determination of the purity of fipronil

Dunnart liver, brain, plasma and fat tissue samples were weighed and homogenised in a Tissuelyser II homogeniser (Qiagen). Samples larger than 0.3 g (liver) were homogenised in a stainless steel 25 mL grinder (Retsch) with a 20 mm stainless steel ball and samples smaller than 0.3 g were homogenised in 2 mL disposable centrifuge tube with a 6 mm diameter stainless steel ball. For every 0.2 g of sample weight, 1 mL of acetonitrile (ACN) with 1% acetic acid (AA) was added. Liver, brain and plasma were homogenised for 3 min at 20 Hz and fat tissue for 9 min at 20 Hz, all at (or close to) −20 °C. Homogenised samples were transferred into disposable 15 mL centrifuge tubes to which 0.5 g of MgSO_4_/NaOAC (4:1 ratio) was added for every 1 mL of ACN +1% AA. Samples were vortexed for 1 min and centrifuged for 4 min at 4000 × *g*. Supernatant (1 mL) was transferred into a 2 mL centrifuge tube with 0.3 g of QuEChERS Dispersive Solid Phase Extraction (1200 mg MgSO_4_, 400 mg primary secondary amine, 400 mg C18, 400 mg graphitised carbon black; LECO Cat. No. 26222-248). The sample was then vortexed for 1 min and centrifuged for 4 min at 16,000 × *g*. Supernatant (200 μL) was then transferred into a 2 mL glass vial with a 250 µl glass insert. Samples were kept at 4 °C during assay.

Samples were analysed on an Agilent 6490 Triple Quad LCMS. Solvents A: H_2_O + 5 mM ammonium formate + 0.2% formic acid. Solvent B: 90% methanol +10% H_2_O + 5 mM ammonium formate + 0.2% formic acid. A Poroshel 120 EC C18 2.7 µm (2.1 × 50 mm) column (InfinityLab) was used and analytes were eluted using a flow rate of 0.2 mL min^−1^ with the following gradient: 1 min at 70% B, 1–10 min 70 to 90% B, 10–11 min 90% B. The volume of injected sample was 1 µL. Fipronil desulfinyl (hereafter referred to as fip-desulfinyl, retention time (RT) 3.2 min), fipronil (RT 3.7 min), fipronil sulfide (hereafter referred to as fip-sulfide, RT 3.9 min) and fip-sulfone (RT 4.5 min) residues were analysed in negative ion mode and were confirmed by their three most abundant product ions at optimised collision energies.

All standards of fipronil and fipronil derivatives were purchased from Sigma Aldrich. A calibration curve was produced using 0.001, 0.01, 0.1, 1 and 10 µg/mL. Standards were prepared fresh and read before, in the middle and at the end of the sample batch. A positive control containing 0.01 µg/mL of fipronil and derivatives and a negative control (ACN +1% AA) was run every three injections to ensure no carry over from previous samples and consistency of quantification. Positive controls contained 0.01 µg/mL of fipronil and derivatives, and negative controls (ACN +1% AA) were run every three samples.

## Results

### Determination of acute oral toxicity

Estimates of the median lethal dose values calculated by the AOT software (Organisation for Economic Cooperation and Development 2001) for male (see Table 1 for dose progression) and female (see Table 2 for dose progression) *S. macroura* were 990 mg kg^−1^ (95% CI = 580.7–4770 mg kg^−1^) and 270.4 mg kg^−1^ (95% CI = 0–>20,000 mg kg^−1^), respectively. The difference in acute oral toxicity values between sexes was potentially influenced by the advanced age of two of the females used in the study (animal ID 016 and 001). Being two of the original field caught animals for the captive breeding colony, these female dunnarts were greater than 12 months older than any other female used in the trial (Table 2). Consequently, the median lethal dose for fipronil was further modelled to assess dunnart responses to fipronil using the following assumptions;

- death at 2000 mg kg^−1^,
- survival at 500 mg kg^−1^, and
- a differential response (both survival and death) at 990 mg kg^−1^.

This modelling revealed median lethal dose estimates for female *S. macroura* of 669.1 mg kg^−1^ (95% CI = 550–990 mg kg^−1^; assuming death at 990 mg kg^−1^) and 990 mg kg^−1^ (95% CI = 544.7–1470 mg kg^−1^; assuming survival at 990 mg kg^−1^.

### Signs of intoxication

Toxicological signs observed following pesticide exposure included piloerection, withdrawal, eye closure, shivering and, intermittently, a lack of response to disturbance. In dunnarts receiving higher doses (e.g., >550 mg kg^−1^), it was not until approximately 24 h after exposure that more severe signs typical of fipronil toxicity, such as tremors and convulsions were observed. The signs of intoxication displayed by each dunnart were video recorded and a full quantitative analysis will be presented in a subsequent publication.

### Dunnart body mass

**Fig. 2:**
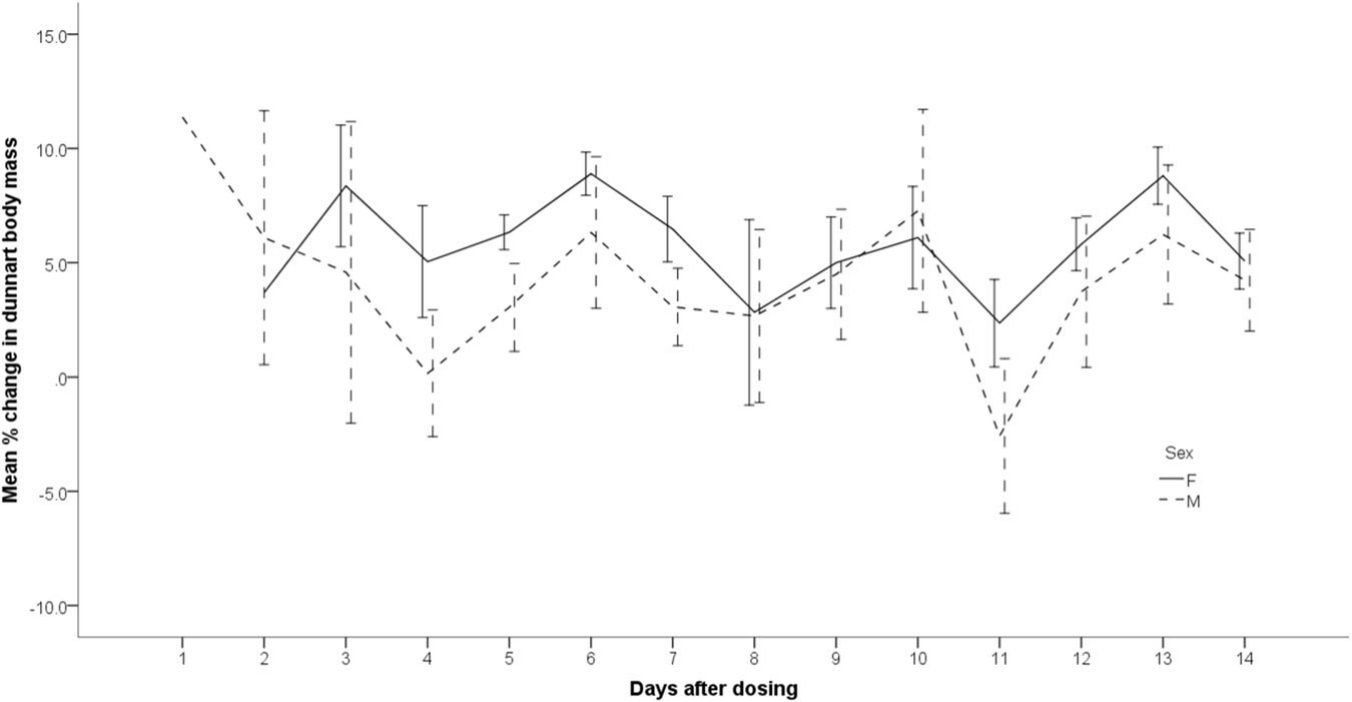
Mean percentage change in dunnart body mass after exposure to fipronil by gavage for all dose levels in those dunnarts not receiving a lethal dose. Error bars represent ±1 standard error and are offset for clarity

Changes in dunnart body mass after exposure show high variability but no discernable pattern (Fig. 2). No statistically significant change in body mass was detected for either males (*t*_0.05(2)3_: *p* = 0.283) or females (*t*_0.05(2)8_; *p* = 0.035) after pesticide exposure using pooled dose data for those dunnarts not receiving a lethal dose.

### Time to death for dunnarts receiving a fatal dose

As only six deaths (two males and four females) occurred within the 48 h time limit placed on the determination of acute oral toxicity, across a range of dose levels from 99 to 2000 mg kg^−1^ (Tables 1 and 2), insufficient data existed for a statistical examination of trends concerning the time to death for dunnarts receiving a lethal dose. From the limited data available, time to death tended to decline with increasing dose greater than 175 mg kg^−1^.

### Residues of fipronil and its metabolites in tissues

**Fig. 3:**
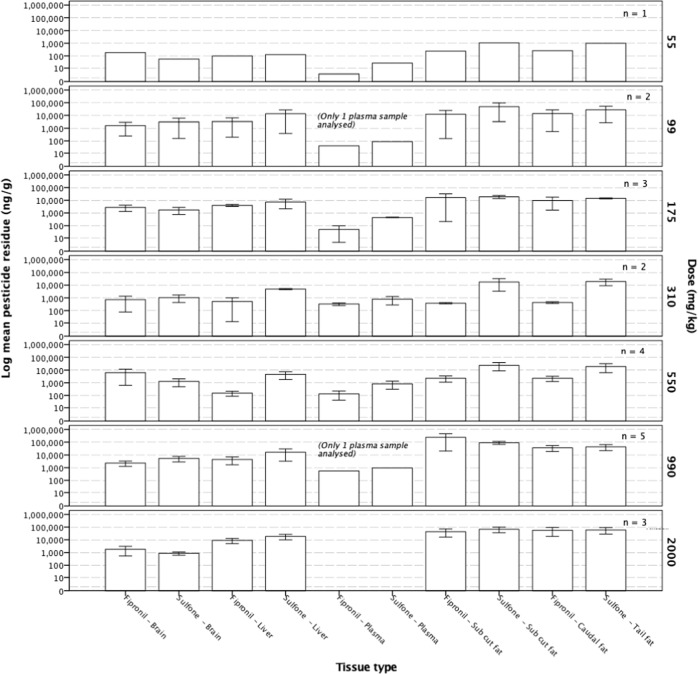
Fipronil and sulfone residues in *Sminthopsis macroura* (Gould 1844) brain, liver, plasma and subcutaneous and tail fat tissue (ng g^−1^) per administered dose (mg kg^−1^) for all animals (*n* = 18). Error bars represent ±1 standard error

**Fig. 4:**
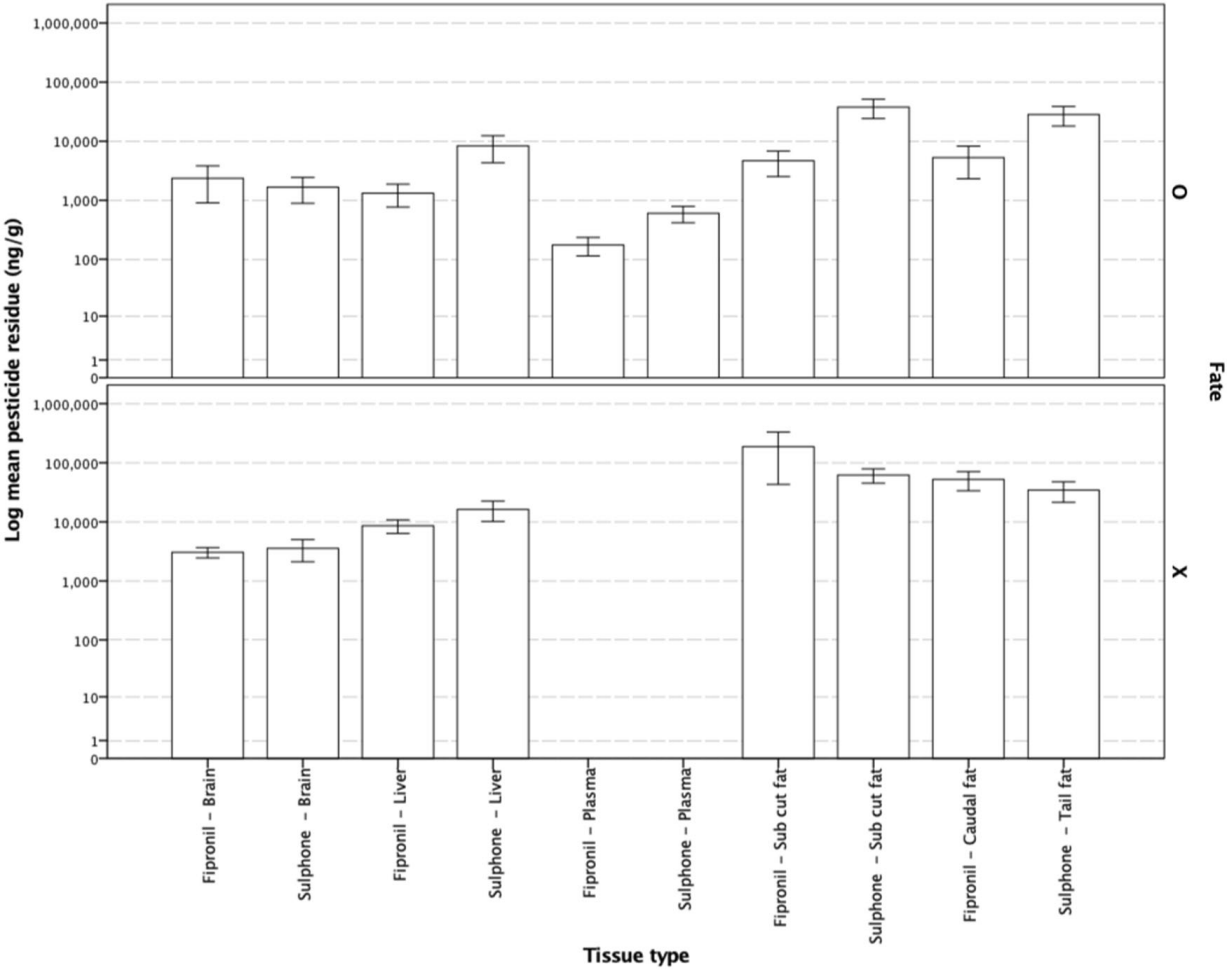
Mean fipronil and sulfone residue levels in *Sminthopsis macroura* (Gould 1844) brain, liver, plasma and subcutaneous and tail fat (ng g^−1^) in animals either surviving (fate = O, *n* = 12) or not surviving (fate = X, *n* = 6) a given dose at 48 h post-exposure. Error bars represent ±1 standard error

Dunnarts given doses of either 990 or 2000 mg kg^−1^ had higher tissue levels of both the parent, fipronil, and the oxidative metabolite, fip-sulfone, in subcutaneous and caudally stored fat samples, although no discernible pattern associating increased tissue residues with an increasing administered dose was evident. Fipronil and fip-sulfone residues were present at very low concentrations in liver, brain and plasma samples taken from dunnarts across all doses, although there is a notable absence of fipronil and fip-sulfone residues in plasma from the highest dose administered (Fig. 3). Dunnarts not surviving the administered dose had higher levels of the parent compound, fipronil, and the oxidative metabolite, fip-sulfone, in liver tissue but similar levels in brain tissue. These dunnarts had higher levels of both fipronil and fip-sulfone in both the subcutaneous and tail fat, indicating that the fip-sulfone is being produced and rapidly, given the time course of the current study, stored in adipose tissues (Fig. 4).

**Fig. 5:**
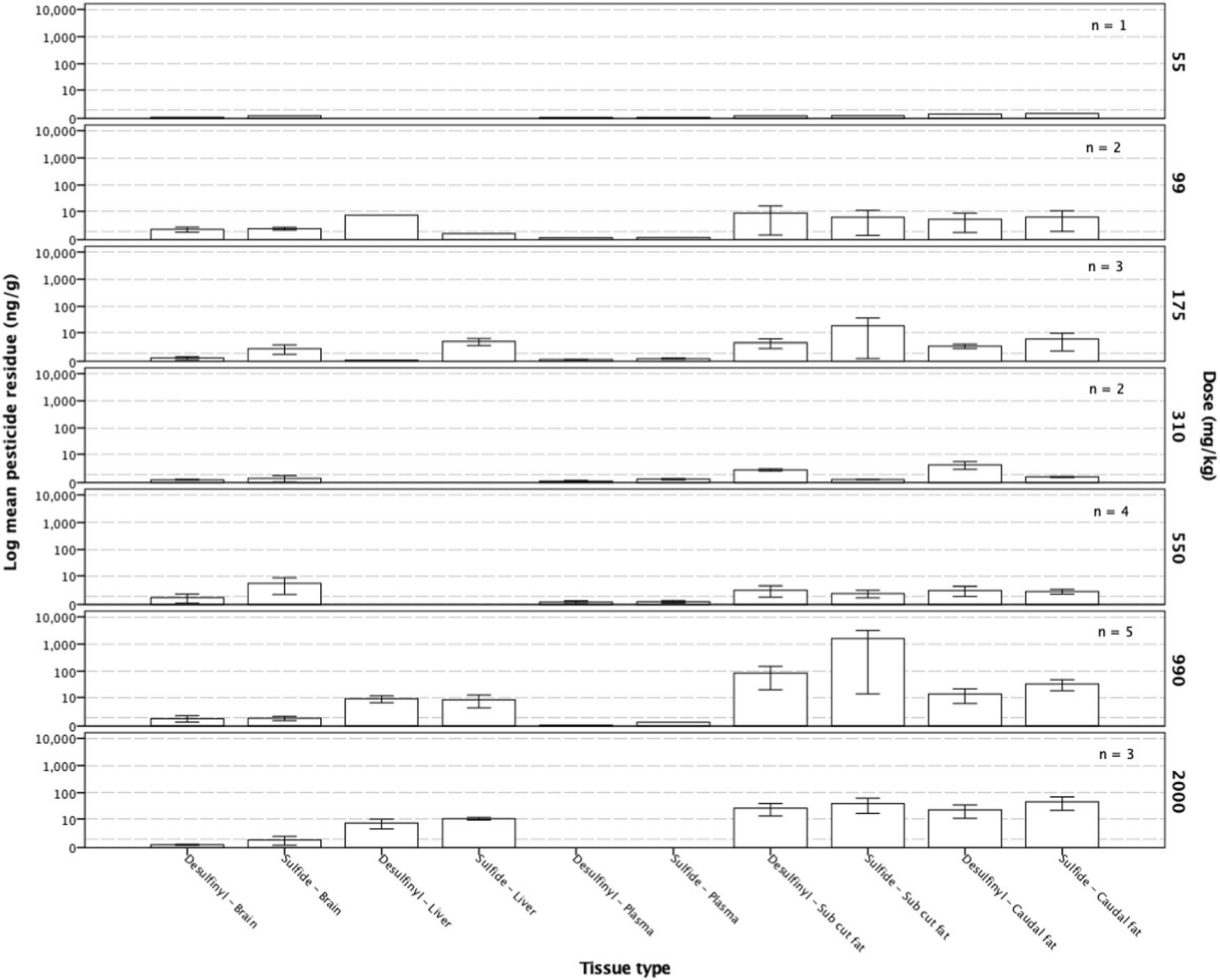
Mean desulfinyl and sulfide metabolite residue levels in *Sminthopsis macroura* (Gould 1844) brain, liver, plasma and subcutaneous and tail fat (ng g^−1^) tissue per administered dose (mg kg^−1^) for all animals (*n* = 18). Error bars represent ±1 standard error

**Fig. 6:**
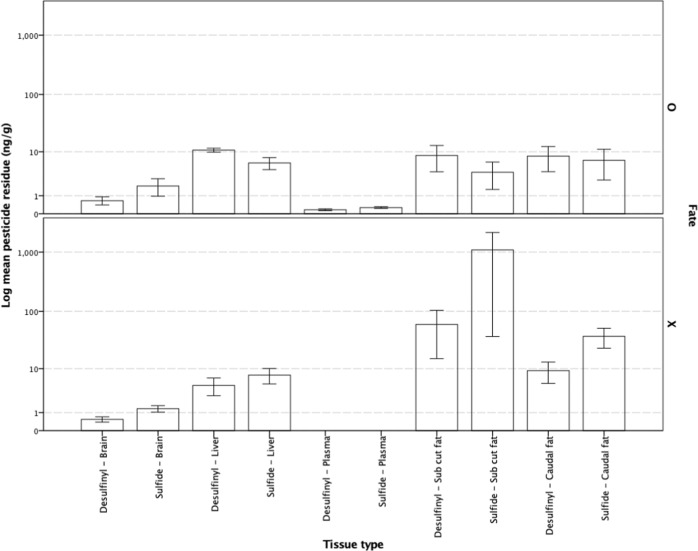
Mean desulfinyl and sulfide metabolite residue levels in *Sminthopsis macroura* (Gould 1844) brain, liver, plasma and subcutaneous and tail fat (ng g^−1^) in animals either surviving (fate = O, *n* = 12) or not surviving (fate = X, *n* = 6) a given dose. Error bars represent ±1 standard error

The lowest administered dose of 55 mg kg^−1^ (*n* = 1) resulted in the detection of very low fip-desulfinyl and fip-sulfide residue levels in brain, liver and adipose tissues. Similarly, detectable residues were also very low in subcutaneous and caudal fat samples but consistent across the remaining doses, again with the exception of plasma (Fig. 5). Comparatively high levels of the fip-sulfide metabolite were also seen in the subcutaneous fat sampled from dunnarts not surviving a given dose. Brain, liver and plasma tissues from dunnarts surviving the dose contained very little, if any, fip-desulfinyl and fip-sulfide metabolite residues. However, the few that did not survive dosing contained higher concentrations of these metabolites in subcutaneous fat (range = 5.91–6354.34 μg kg^−1^), with smaller amounts stored in tail fat (range = 2.00–85.78 μg kg^−1^) (Fig. 6).

**Fig. 7:**
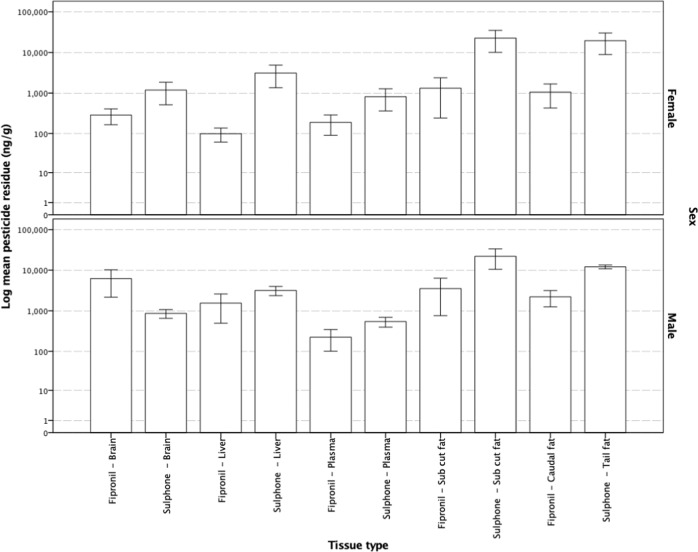
Mean fipronil and sulfone resides in male (*n* = 7) and female (*n* = 11) *Sminthopsis macroura* (Gould 1844) brain, liver, plasma and subcutaneous and tail fat (ng g^−1^). Error bars represent ±1 standard error

**Fig. 8:**
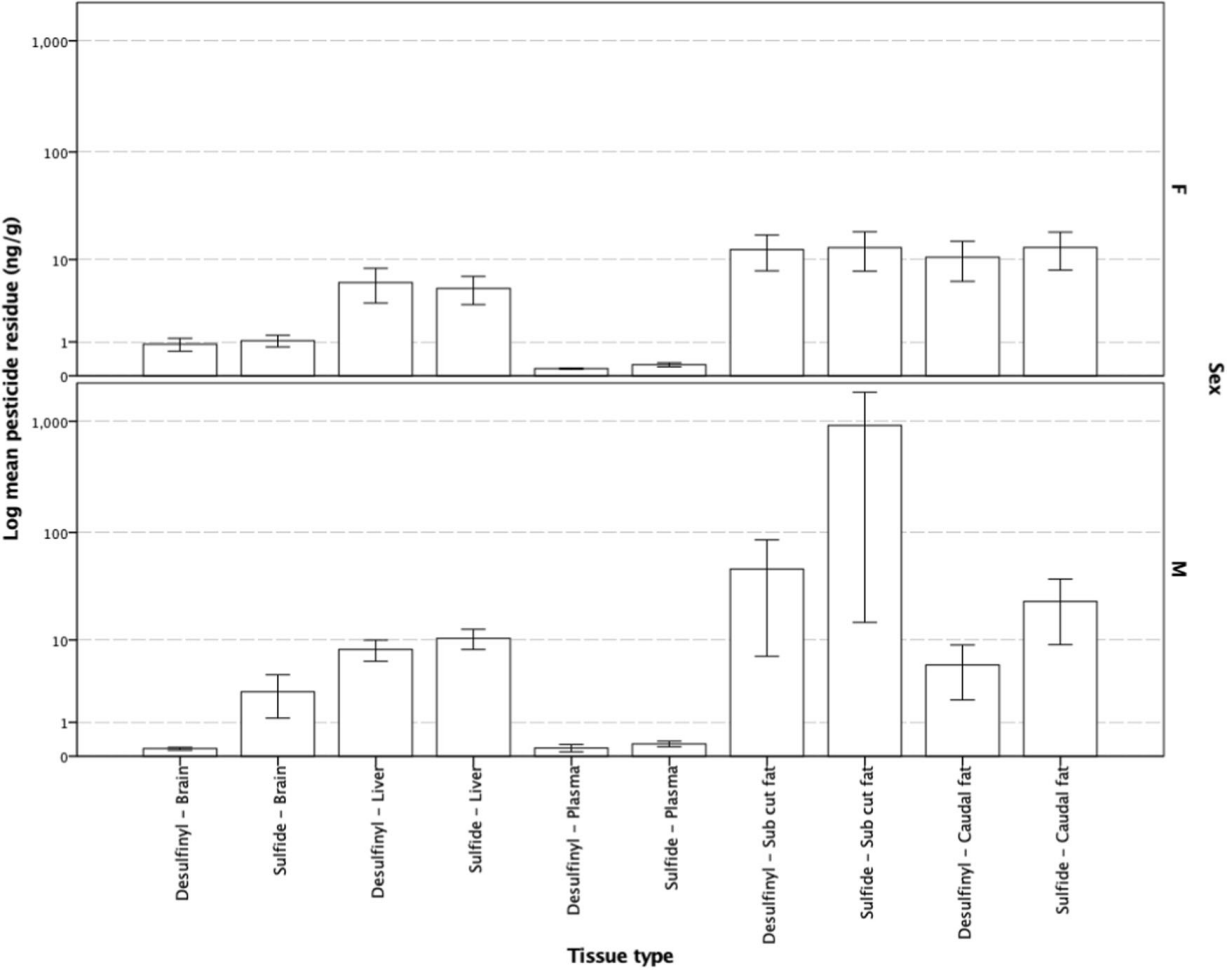
Mean desulfinyl and sulfide metabolite resides in male (*n* = 7) and female (*n* = 11) *Sminthopsis macroura* (Gould 1844) brain, liver, plasma and subcutaneous and tail fat (ng g^−1^). Error bars represent ±1 standard error

Mean fipronil and fip-sulfone tissue levels were similar in male and female dunnarts with maximal residues being detected in subcutaneous and caudally stored fat. Both male and female dunnarts demonstrated an equal propensity to store both fipronil and fip-sulfone in subcutaneous and tail fat reserves. Males had comparatively higher levels of fipronil in brain tissue than females, although sulfone in the brain and liver tissues sampled were similar (Fig. 7). While male dunnarts showed fip-sulfide and fip-desulfinyl residues in subcutaneous and caudally stored fat, female residue levels were extremely low (Fig. 8).

## Discussion

### Median lethal dose

Both genders of *S. macroura* tested in the current study were significantly less sensitive to fipronil than the only other mammals tested, *M. musculus* (L. 1758; 94 mg kg^−1^) and *Rattus norvegicus* (Birkenhout 1769; 97 mg kg^−1^) (Food and Agriculture Organisation of the United Nations 1997) in the literature to date. This result directly contrasts with a 10–14-fold difference in acute oral toxicity for both dunnart species (*S. crassicaudata* = 129 mg kg^−1^ CI = 74.2–159.0; *S. macroura* = 97 mg kg^−1^ CI = 88.3–120.0) to the organophosphorous pesticide, fenitrothion, when compared to *M. musculus* (1100–1400 mg kg^−1^), using the same technique for the resolution of median lethal dose estimates (Story et al. 2011). Whilst the two chemicals mentioned above exert their influence on different physiological pathways, the significant differences in patterns of acute oral toxicity compound the lack of acute oral vertebrate toxicological data thereby reducing the predictive value of pesticide risk assessments for endemic Australian vertebrates based on data from non-native species.

Current risk assessment frameworks for pesticides generally use, in part, the lowest median lethal dose for mammals to assess hazard of a chemical (Newman 2015). Increasingly, median lethal dose estimates, either LD_50_ or LC_50_ data, obtained from chemical exposure studies can be incorporated into species sensitivity distributions (SSDs) to comparatively assess toxicity and derive hazard threshold values (Posthuma et al. 2002). However, the generation of a distribution using three data points, while possible with the assistance of extrapolation factors (as outlined in Posthuma et al. (2002)), is less likely to provide a robust representation of the desired risk thresholds (e.g., HD_05_) rendering the estimation of safe residue levels problematic. Recent research has highlighted a similar problem in relation to the avian acute oral toxicity profile of fipronil. While previous risk assessments for this pesticide have cited a primarily bimodal toxicological profile with a highly sensitive species at one end (the northern bobwhite, *Colinus virginianus* L. 1758; LD_50_ = 11.3 mg kg^−1^) and an extremely tolerant species at the other (the mallard, *Anas platyrhynchos* L. 1758; LD_50_ = 2150 mg kg^−1^*)*, Kitulagodage et al. demonstrated that, by testing other species, the acute oral toxicity of fipronil fits a distribution similar to that of other pesticides and is grouped along avian orders (Kitulagodage 2011; Kitulagodage et al. 2011b).

The advantages of using the UDP protocol for the derivation of median lethal doses over the traditional LD_50_ assessment techniques are well established (Newman 2013; Story et al. 2011). Specifically, a reduction in the number of individuals required to resolve an estimate of median lethal dose is desirable from an animal ethics perspective, particularly if the use of other chemical impact metrics (e.g., quantitative structure-activity relationships, QSARs) to assess the potential sensitivity of untested species to a pesticide are precluded due to a lack of data (Story et al. 2011). Additionally, in quantifying a median lethal dose the UDP method uses far fewer animals than conventional toxicity tests while the results are directly comparable to other testing protocols, thus allowing a comparison of pesticide sensitivity of an Australian metatherian species with non-native eutherian mammals (Story et al. 2011).

The assessment of agricultural and veterinary chemicals for registration in Australia is a process that is evolving over time as both the amount of data submitted to support registrations increases and assessment methodologies and detection levels improve (Hyman 1997). If the use of SSDs to assess protection thresholds in relation to Australian endemic species is to continue, then further sensitivity research will be required to circumvent the need to extrapolate from a narrow range of organisms tested under standard laboratory conditions to free-living populations or ecosystems. The results of the present study show the limitations of this approach and highlight the importance of evaluating the effects of pesticides on non-target species that are likely to be exposed, particularly when these species are phylogenetically distinct from those used in previous studies of pesticide sensitivity originating in North America or the European Union.

### Fiprole (fipronil and metabolite) residues in tissues and body mass

The use of the UDP methodology to quantify a median lethal dose unavoidably results in very small experimental groups, sometimes *n* = 1, thereby resulting in secondary data sets, such as residue loads from tissue samples, that are unsuitable for statistical analysis. Despite this limitation, the current study quantified fiprole residue levels in kidney, liver, plasma, brain and caudal and subcutaneous adipose tissue samples taken from individual dunnarts at either the time of death or at the end of the 14 day post-dose observation period. Obviously, these results need to be viewed with a great deal of circumspection. However, we report these results from the current study as a precursory dataset to maximise the amount of information derived and to better inform a subsequent study into the comparative metabolic fate of fipronil in two similar-sized, but systematically divergent species, *M. musculus* (eutherian) and *S. macroura* (metatherian) accepting the abovementioned limitations.

Studies investigating the biotransformation of fipronil in rats (Food and Agriculture Organisation of the United Nations 1997) have quantified three primary metabolites after hepatic transformation of the parent compound fipronil (Fig. 1). Of these metabolites, the fip-sulfone and fip-desulfinyl were of toxicological concern in previous studies. The oxidative fip-sulfone metabolite has a six-fold higher binding affinity for the postsynaptic GABA receptor (Hainzl et al. 1998) and metabolism of the parent compound to this derivative adds synergistically to the overall toxicity of a fipronil-based formulation in pesticide-exposed birds (Kitulagodage et al. 2011b). Moreover, avian studies have demonstrated that inclusion of fip-sulfone residues in a regression analysis of post-exposure body mass loss provided a much better fit than regressions comparing loss of body mass with the parent compound, fipronil, alone in brain, liver and adipose tissues (Kitulagodage et al. 2011b). The overlap between symptoms of intoxication, the time course of fip-sulfone residues in brain, liver and adipose tissue, lack of post-dose feeding activity and subsequent weight loss in dosed birds provided insight into an observed selective toxicity to the three galliform species tested (Kitulagodage 2011; Kitulagodage et al. 2011b).

In the current study, fipronil and fip-sulfone residues were more prominent at the higher doses administered (e.g., 990 and 2000 mg kg^−1^) with the residue load occurring in subcutaneous and caudally stored fat, liver and brain, in descending order of magnitude. Slightly higher levels of fipronil were present in male (versus female) brains at the time of analysis, but very little difference existed between either fipronil or fip-sulfone levels in either subcutaneous or tail fat and plasma. Dunnarts not surviving an administered dose had higher fipronil and fip-sulfone levels in adipose tissue, liver and brain (Fig. 4). However, in dunnarts surviving a given dose, very little fipronil and fip-sulfone residues were detected in plasma, bringing into question whether the use of fipronil residue in plasma is suitable as a biomarker of pesticide exposure in wildlife monitoring studies. Detection of fip-sulfone in the liver and adipose tissues of males and females across doses indicates the metabolism of fipronil to the fip-sulfone metabolite occurs readily and the levels detected, in addition to low levels of this metabolite finding its way to brain tissue, as well as an absence of weight loss in dunnarts surviving the administered dose, is contrary to the findings in the abovementioned avian studies (Kitulagodage et al. 2011b). Further research into the metabolic fate of this pesticide in marsupials is required to better elucidate the role of the fip-sulfone metabolite in determining the overall toxicity of fipronil-based pesticide formulations, as seems to be the case in more sensitive avian orders.

Fip-desulfinyl is generally considered to be a photolytic breakdown product and not a metabolite as such. In the current study, analysis detected generally low levels of this compound (range = 0–46.07 ng g^−1^ with one male dunnart (dose = 99 mg kg^−1^) returning an outlier value of 281.89 ng g^−1^ in adipose tissue) and due to its toxicological significance, we have reported these results. Fip-desulfinyl is considered of high toxicity with an acute oral LD_50_ of 15 (males)–18 (females) mg kg^−1^ for *M. musculus* (Food and Agriculture Organisation of the United Nations 1997). When administered orally to mice, the fip-desulfinyl metabolite has been shown to decrease body weight at doses of 30 and 60 mg kg^−1^, whereas a lower dose of 3 mg kg^−1^ increased motor activity, irritability and aggression with convulsions also observed (Food and Agriculture Organisation of the United Nations 1997). Although present in small quantities, presumably as a result of photolytic breakdown of the dosing formulation immediately after preparation and prior to ingestion, its acute toxicity would necessitate its inclusion in residue analysis for any future laboratory, or field-based, trials investigating wildlife impacts. Higher levels of the fip-sulfide metabolite (range = 0–85.78 ng g^−1^ within the same male dunnart as above (dose = 99 mg kg^−1^) with an outlier value of 6345.34 ng g^−1^) were also found in adipose tissues of pesticide-exposed dunnarts. The higher LD_50_ values for this compound reported for mice (69 (males) and 100 (females) mg kg^−1^ (Food and Agriculture Organisation of the United Nations 1997)) indicates a moderate toxicity for this species, with similar toxicological signs as those reported for the other breakdown products, fip-sulfone and fip-desulfinyl, as well as the parent compound, fipronil.

The Australian arid zone is characterised by low productivity and highly variable rainfall (Stafford-Smith and Morton 1990). Species inhabiting these environments have evolved a range of adaptations which assist them in coping with the inconsistent, and often sparsely distributed resources—such as the ability for rapid, long-range movement enabling animals to access areas of recent rainfall and capitalise on the increase food resources (Dickman et al. 1995; Letnic and Dickman 2005). The Dasyuridae caudally store fat to provide an energy reserve that can be utilised during times of resource limitation (Morton and Dickman 2008a, b). The ability for lipophilic xenobiotic compounds, such as agricultural pesticides and their toxic metabolites, to be stored along with these fat reserves has the potential to ensure that pesticide residues remain biologically available by being constantly metabolised as dunnarts utilise caudally stored fat to maintain the energetic resources necessary for sustaining daily life during times of drought. Moreover, as conventional toxicity testing used for chemical risk assessments generally defines exposure times for the determination of median lethal dose values to quantify mortality (Newman 2015), the tendency for toxic substances to be stored in adipose tissue and later metabolised when animals are facing resource limitations, extends the exposure period for chemicals significantly beyond, for example, either the 48 h acute oral toxicity test limit or the 30 d reproductive test limit more commonly used in pesticide risk assessments (Buttemer et al. 2008; Story et al. 2016).

## Conclusions

The scarcity of information quantifying the responses of Australian endemic species to pesticides impedes the development of biologically relevant risk assessments for the registration of chemicals in Australia. The lack of sensitivity to fipronil displayed by *S. macroura*, as measured by acute oral toxicity, directly contrasts with the increased sensitivity (10–14 fold) of the same species to another locusticide, fenitrothion (Story et al. 2011), highlighting the need for a better understanding of the biochemical pathways responsible for any species susceptibility to xenobiotics and thereby increasing the predictive value of risk assessments. Additional studies are now required to better understand the metabolic fate and biochemical parameters responsible for pesticide metabolism in mammals, particularly when the active ingredient of pesticide formulations can produce toxic metabolites. Finally, while the relatively high median lethal dose values quantified here would suggest a minimal impact of pesticide exposure on the species tested, no information quantifying the pesticide exposure of *S. macroura* in situ exists. Clearly, more research into dietary and non-dietary pesticide exposure pathways and residue loads are required to better inform environmental impact assessments.

## References

[CR1] Balanca G, de Visscher M-N (1997). Effects of very low doses of fipronil on grasshoppers and non-target insects following field trials for grasshopper control. Crop Prot.

[CR2] Barnthouse LW, Munns WR, Sorensen MT (2008). Population-level ecological risk assessment.

[CR3] Bijleveld van Lexmond MFIJ, Bonmatin J-M, Goulson D, Noome DA (2014). Worldwide integrated assessment on systemic pesticides. Environ Sci Pollut Res.

[CR4] Bobe A, Cooper J-F, Coste CM, Muller M-A (1998). Behaviour of fipronil in soil under Sahelian Plain field conditions. Pestic Sci.

[CR5] Bruce RD (1985). An Up-And-Down procedure for acute toxicity testing. Fundam Appl Toxicol.

[CR6] Buttemer WA, Story PG, Fildes KJ, Baudinette RV, Astheimer LB (2008). Fenitrothion, an organophosphate, affects running endurance but not aerobic capacity in fat-tailed dunnarts (*Sminthopsis crassicaudata*). Chemosphere.

[CR7] Chagnon M, Kreutzweiser D, Mitchell EAD, Morrissey CA, Noome DA, van der Sluijs JP (2014). Risks of large-scale use of systemic insecticides to ecosystem functioning and services. Environ Sci Pollut Res.

[CR8] Dickman CR, Predavec M, Downey F (1995). Long range movements of small mammals in arid Australia: implications for land management. J Arid Environ.

[CR9] Environment Australia (1998). Consolidated Environmental Assessment Report.

[CR10] Food and Agriculture Organisation of the United Nations (1997). Fipronil. Toxicological and environmental evaluations.

[CR11] Hainzl D, Cole LM, Casida JE (1998). Mechanisms for selective toxicity of fipronil insecticide and it’s sulfone metabolite and desulfinyl photoproduct. Chem Res Toxicol.

[CR12] Holder PJ, Jones A, Tyler CR, Cresswell JE (2018). Fipronil pesticide as a suspect in historical mass mortalities of honey bees. Proc Natl Aca Sci USA.

[CR13] Hyman M (1997) Pesticide related risks – key issues for the Australian environment. In BoR Sciences (ed) Australian National Pesticide Risk Reduction Workshop, 16–18 April, 1997, Canberra, Australian Capital Territory, Australia, Canberra, Australia, pp 33–38

[CR14] IBM SPSS (2017) IBM SPSS Statistics Version 27. Armonk, New York, USA

[CR15] Kitulagodage M (2011). Impact of fipronil, a new generation pesticide, on avian development and health.

[CR16] Kitulagodage MK, Astheimer LB, Buttemer WA (2008). Diacetone alcohol, a dispersant solvent, contributes to acute toxicity of a fipronil-based insecticide in a passerine bird. Ecotoxicol Environ Saf.

[CR17] Kitulagodage MK, Buttemer WA, Astheimer LB (2011). Adverse effects of fipronil on avian reproduction and development: maternal transfer of fipronil to eggs in zebra finch *Taeniopygia guttata* and in ovo exposure in chickens *Gallus domesticus*. Ecotoxicology.

[CR18] Kitulagodage MK, Isanhart J, Buttemer WA, Hooper MJ, Astheimer LB (2011). Fipronil toxicity in northern bobwhite quail *Colinus virginianus*: Reduced feeding behaviour and sulfone metabolite formation. Chemosphere.

[CR19] Letnic M, Dickman CR (2005). The responses of small mammals to patches regenerating after fire and rainfall in the Simpson Desert, central Australia. Austral Ecol.

[CR20] Lipnick R, Cotruvo J, Hill R, Bruce R, Stitzel K, Walker A, Chus I, Goddard M, Segal L, Springer J, Myers R (1995). Comparison of the up-and-down, conventional LD50 and fixed-dose acute toxicity procedures. Food Chem Toxicol.

[CR21] Morton SR, Dickman CR, van Dyck S, Strahan R (2008). Fat-tailed dunnart, *Sminthopsis crassicaudata* (Gould 1844). The Marsupails of Australia.

[CR22] Morton SR, Dickman CR, van Dyck S, Strahan R (2008). Stripe-faced dunnart, *Sminthopsis macroura* (Gould 1845).. The Mammals of Australia.

[CR23] Newman MC (2013). Quantitative ecotoxicology.

[CR24] Newman MC (2015). Fundamentals of ecotoxicology. The science of pollution.

[CR25] Organisation for Economic Cooperation and Development (2001). Acute oral toxicity—up and down procedure. OECD Guideline 425.

[CR26] Peveling R, Attington S, Langewald J, Ouambama Z (1999). An assessment of the impact of biological and chemical grasshopper control agents on ground-dwelling arthropods in Niger, based on presence/absence sampling. Crop Prot.

[CR27] Peveling R, McWillian AN, Nagel P, Rasolomanana H, Raholijaona, Rakotomianina L, Ravoninjatovo A, Dewhurst CF, Gibson G, Rafanomezana S, Tingle CCD (2003). Impact of locust control on harvester termites and endemic vertebrate predators in Madagascar. J Appl Ecol.

[CR28] Posthuma L, Suter GW, Trass TP (2002). Species sensivity distributions in ecotoxicology. Environmental and ecological risk assessment.

[CR29] Selwood L, Cui S (2006). Establishing long-term colonies of marsupials to provide models for studying developmental mechanisms and their application to fertility control. Aust J Zool.

[CR30] Smith PN, Afzal M, Al-Hasan R, Bouwman H, Castillo LE, Depledge M, Subramanian M, Dhananjayan V, Fossi C, Kitulagodage MK, Kylin H, Law R, Marsili L, O’Hara T, Spinola M, Story PG, Goddard-Codding C, Kendall RJ, Lacher TE, Cobb GP, Cox SB (2010). Global perspectives on wildlife toxicology: emerging issues. Wildlife toxicology: emerging contaminant and biodiversity issues.

[CR31] Stafford-Smith DM, Morton SR (1990). A framework for the ecology of arid Australia. J Arid Environ.

[CR32] Steinbauer MJ, Peveling R (2011). The impact of the locust control insecticide fipronil on termites and ants in two contrasting habitats in northern Australia. Crop Prot.

[CR33] Story PG (2015). Sensitivity of the dasyurids, *Sminthopsis crassicaudata* (Gould 1844) and *S. macroura* (Gould 1845) to the organophosphorus insecticide, fenitrothion, and its impact on locomotory and thermogenic performance in S. macroura.

[CR34] Story PG, French K, Astheimer LB, Buttemer WA (2016). Fenitrothion, an organophosphorus insecticide, impairs locomotory function and alters body temperatures in *Sminthopsis macroura* (Gould 1845) without reducing metabolic rates during running endurance and thermogenic performance tests. Environ Toxicol Chem.

[CR35] Story PG, Hooper MJ, Astheimer LB, Buttemer WA (2011). Acute oral toxicity of an organophosphorus pesticide, fenitrothion, to fat-tailed and stripe-faced dunnarts and its significance for risk assessments in Australia. Environ Toxicol Chem.

[CR36] Story PG, Walker PW, McRae H, Hamilton JG (2005). A case study of the Australian Plague Locust Commission and environmental due diligence: why mere legislative compliance is no longer sufficient for environmentally responsible locust control in Australia. Integr Environ Assess Manag.

[CR37] Tingle CCD, Rother JA, Dewhurst CF, Lauer S, King WJ (2000). Health and environmental effects of fipronil.

[CR38] Tomlin CDS (2006). The pesticide manual, a world compendium.

[CR39] van Dyck S, Strahan R (2008). The mammals of Australia.

[CR40] van Straalen NM, Newman MC (2002). Theory of ecological risk assessment based on species sensitivity distributions. Species sensitivity distributions in ecotoxicology.

[CR41] Walker PW, Story PG, Hose GC (2016). Comparative effects of pesticides, fenitrothion and fipronil, applied as ultra-low volume formulations for locust control, on non-target invertebrate assemblages in Mitchell grass plains of south-west Queensland, Australia. Crop Prot.

